# Association between Asian dust exposure and respiratory function in children with bronchial asthma in Nagasaki Prefecture, Japan

**DOI:** 10.1186/s12199-020-00846-9

**Published:** 2020-03-04

**Authors:** Takahiro Nakamura, Yuji Nishiwaki, Kunio Hashimoto, Ayano Takeuchi, Tasuku Kitajima, Kazuhiro Komori, Kasumi Tashiro, Hideki Hasunuma, Kayo Ueda, Atsushi Shimizu, Hiroshi Odajima, Hiroyuki Moriuchi, Masahiro Hashizume

**Affiliations:** 1 Department of Environmental and Occupational Health, School of Medicine, Toho University, Tokyo, Japan; 2 Department of Paediatrics, Graduate School of Biomedical Sciences, Nagasaki University, Nagasaki, Japan; 3 Department of Preventive Medicine and Public Health, School of Medicine, Keio University, Tokyo, Japan; 4 Department of Paediatrics, Nagasaki Goto Chuoh Hospital, Nagasaki, Japan; 5 Department of Paediatrics, Nagasaki Kamigoto Hospital, Nagasaki, Japan; 6 Department of Paediatrics, Isahaya General Hospital, Nagasaki, Japan; 7 Center for Environmental Information Science, Tokyo, Japan; 8 Environmental Health Sciences, Graduate School of Global Environmental Studies, Kyoto University, Kyoto, Japan; 9 Center for Regional Environmental Research, National Institute for Environmental Studies, Ibaraki, Japan; 10 Department of Paediatrics, Fukuoka National Hospital, Fukuoka, Japan; 11 Department of Global Health Policy, School of International Health, Graduate School of Medicine, The University of Tokyo, Tokyo, Japan

**Keywords:** Asian dust, Bronchial asthma, Children, Peak expiratory flow rate

## Abstract

**Background:**

Studies on the adverse effects of Asian dust (AD) on respiratory function in children are scarce. The objective of this study was to examine the association between AD and respiratory function by measuring peak expiratory flow rates (PEFRs) in asthmatic children.

**Methods:**

The study was carried out from March to May from 2014 through 2016. One hundred ten children with bronchial asthma were recruited from four hospitals in the Goto Islands and south Nagasaki area in Nagasaki prefecture. The parents were asked to record their children’s PEFRs every morning/evening and clinical symptoms in an asthma diary. AD was assessed from light detection and ranging data, and a linear mixed-effects model was used to estimate the effects of AD on daily PEFR. Time-stratified case-crossover analyses were performed to examine the association between AD and asthma attacks defined by reduction levels in PEFR.

**Results:**

AD was detected on 11 days in the Goto Islands, and on 23 days in the south Nagasaki area. After adjusting for age, sex, temperature, and daily oxidants, we found a consistent association between AD and a 1.1% to 1.7% decrease in PEFR in the mornings and a 0.7% to 1.3% decrease in the evenings at a lag of 0 to 5 days. AD was not associated with the number of asthma attacks, respiratory symptoms, or other symptoms at any lag days examined.

**Conclusions:**

Exposure to AD was associated with reduced PEFR, although the effects were not large enough to induce clinically apparent symptoms, in clinically well-controlled asthmatic children.

## Background

Asian dust (AD) originates in the Yellow River basin and deserts of northern China and Mongolia and is carried long distances by prevailing winds [1]. Although this is a natural phenomenon, it is also an environmental problem throughout Northeast Asia. The fundamental cause is deforestation, soil degradation, and desertification and AD contains various toxic pollutants [2, 3]. The type and degree of damage caused by AD depend on the distance from the source of the dust and weather conditions [4]. AD most often affects western areas of Japan from March through May, although it can be observed throughout the year. AD can be monitored both visually and by light detection and ranging (LIDAR), with the latter method being more accurate [5].

Epidemiological studies from East Asia have detected an association between AD and adverse health events, including respiratory and cardiovascular diseases [ 6–8]. Children appear to be more vulnerable than adults, both in terms of susceptibility and response to environmental changes, including air pollution [ 9–13]. In particular, children with bronchial asthma are more easily affected by environmental changes [14]. We have previously reported that exposure to AD in Nagasaki was associated with increased emergency department visits by schoolchildren due to asthma attacks and by preschool children due to respiratory diseases [15]. Reduced peak expiratory flow rates (PEFRs) would be expected in advance of emergency hospital visits due to bronchial asthma attacks. In a study of pulmonary function involving daily measurements of PEFR, Hong et al. reported that outdoor particulate concentrations during periods of AD coincided with reduced PEFR in asthmatic children but not in non-asthmatic children [16]. However, data on the effects of AD on this susceptible population are scarce.

The purpose of this study, therefore, was to examine the association of AD with respiratory function measured by PEFR and clinical symptoms in asthmatic children.

## Methods

### Study design and participants

**Fig. 1:**
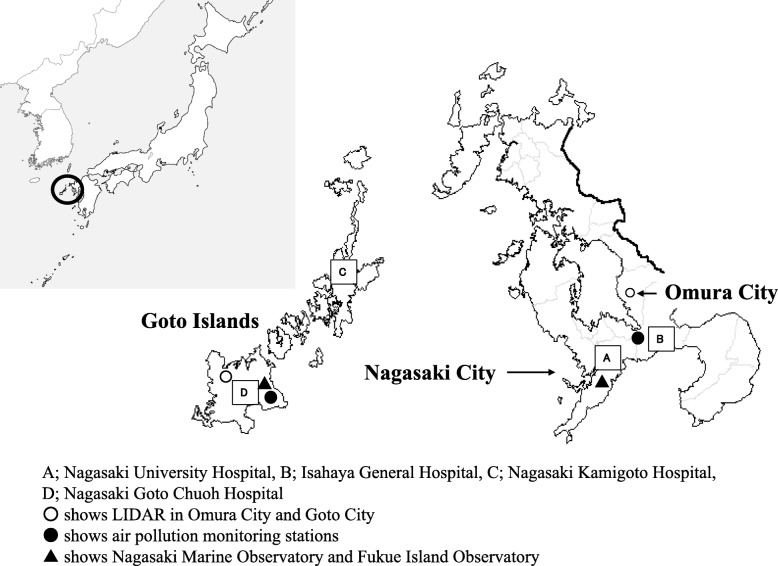
Map of Japan (left) and Nagasaki prefecture (right)

The study cohort was made up of 110 children younger than 15 years of age with bronchial asthma. Participants were recruited from four hospitals in Nagasaki Prefecture, the westernmost prefecture in Japan, from 2014 through 2016. The children had all been diagnosed with bronchial asthma according to the Japanese Pediatric Guideline for the Treatment and Management of Asthma 2012 criteria and had each received regular outpatient follow-up care at one of the hospitals [17]. The first study site was the Goto Islands, and 37 children were recruited from two local hospitals: Nagasaki Kamigoto Hospital and Nagasaki Goto Chuoh Hospital. The second study site was the southern part of Nagasaki Prefecture, where 73 children were recruited from Nagasaki University Hospital and Isahaya General Hospital (Fig. 1). The Goto Islands form an archipelago in the East China Sea, approximately 100 km west of Nagasaki City on the Kyushu mainland. The largest and southernmost of the Goto Islands is Fukue Island.

We designed a 3-year cohort study, with participants recruited each fiscal year. Fifty (45.5%) of the children were followed for 3 years, 31 (28.2%) for 2 years, and 29 (26.4%) for 1 year.

At the entry of the study, we gathered information via a questionnaire on age, sex, height, weight, duration of daily outdoor activities, usage of an air cleaner, and the presence of cohabiting smokers. We also collected data from the children’s physicians regarding asthma severity, long-term medications, and total serum IgE.

### Measurement of AD

As described in our previous studies [15, 18], we used LIDAR data obtained from two installations belonging to the National Institute for Environmental Studies to assess exposure to AD. This LIDAR network can estimate the dust extinction coefficients of non-spherical and spherical components. One installation is in Omura City, approximately 30 km from Nagasaki City, and the other is in the Goto Islands (Fig. 1). LIDAR uses polarized laser light to recognize shape differences, and it can distinguish AD particles from other air pollutants, which are generally spherical. The lowest altitude for which LIDAR data are available is 120–270 m above the ground, but if the lower atmosphere is well mixed, the concentration of AD at that altitude is similar to that at ground level [19]. LIDAR measurements were compiled as total 24-h data (midnight to midnight). AD extinction coefficients larger than 1/km and spherical extinction coefficients larger than 2/km were excluded because these data may be influenced by meteorological factors such as cloud, fog, rain, and snow [18].

In our study, we defined “AD days” when all of the following criteria were met [20].

1. The daily maximum suspended particulate matter (SPM) concentration was over 50 μg/m^3^.
2. The daily maximum AD extinction coefficient as measured by LIDARfrom was more than 0.05/km.
3. The correlation coefficient between hourly AD extinction coefficient and hourly SPM concentration was significant (*p* < 0.01).

For SPM concentrations, we used the daily average of hourly measurements made at one air pollution monitoring station in Isahaya City and at one in the Goto Islands. SPM was defined under the Japanese Air Quality Standard as any particle with an upper 100% cut-off point of 10 μm in aerodynamic diameter.

### Other air pollutants and meteorological data

Data on other air pollutants (sulfur dioxide [SO_2_], nitrogen dioxide [NO_2_], and photochemical oxidants [Ox]) were collected from air pollution monitoring stations in Isahaya city and the Goto Islands. Daily average concentrations of SO_2_ and NO_2_ were calculated from hourly concentrations measured at each station. Ox was defined as any mixture of ozone and other secondary oxidants generated by a photochemical reaction, and it was considered to be a proxy for ozone. Maximum hourly concentrations of Ox measured at each station were used. Data based on fewer than 20 hourly measurements on any 1 day were excluded [18].

Daily temperature and relative humidity data for south Nagasaki and the Goto Islands were obtained from the Japanese Meteorological Agency’s local meteorological observatories in Nagasaki and Fukue Island, respectively. We calculated the daily average temperature and relative humidity from data collected hourly at each observatory (between 00:00 and 23:00) and recorded the maximum and minimum temperatures.

### Asthma diary records of PEFR and clinical symptoms

#### Asthma diary

The parents were asked to record their children’s PEFRs in the asthma diary. They were also asked to record children’s clinical symptoms, which were respiratory symptoms related to asthma attacks (cough, wheezing, shortness of breath, attacks in exercise, and sleep disturbance due to asthma attacks), and any other symptoms affecting the eyes, skin. The use of asthma medications and emergency clinic visits was also recorded.

#### PEFR measurements

In March through May each year during the study period, PEFRs were measured three times each morning and evening before participants inhaled either corticosteroids or β2-agonists. A Wright PEF meter (Clement Clarke International, London, UK) was used for the measurements, and the highest of the three rates measured each morning and evening were recorded. The participants and their parents practiced using the meter to measure PEFR and keeping a diary of measurements for the month before the study commenced (i.e., in February). Asthma attack was defined as mild, moderate, and severe if a reduction of PEFR was less than 80%, 70% to 79%, and below 70%, respectively of the monthly maximum values in each participant [17].

### Statistical analysis

Mann-Whitney *U* tests and Student’s *t* tests were used to compare AD extinction coefficients, spherical extinction coefficients, SPM, other air pollutants, and meteorological data on AD days and non-AD days in both of the study areas. A linear mixed-effects model was used to estimate the effects of AD on daily PEFRs. Effects (PEFR changes) were estimated after adjusting for age, sex, daily mean temperature, daily relative humidity and daily concentration of Ox.

The association between AD exposure and clinical symptoms (respiratory, skin, eye, and nose) and asthma attacks defined by the reduction in PEFR were also examined using a time-stratified case-crossover design. A day on which asthma attack occurred was assigned as the case day, and the same day of the previous or later weeks in the same month of the same year was assigned as control days. For this analysis, we used conditional logistic regression models and the results were expressed as odds ratios (ORs) and 95% confidence intervals (CIs). In the models, temperature and relative humidity were adjusted for. All the covariates were averaged on the case day (lag 0) and the two following days (lag 1 and lag 2). As adjustments by other air pollutants (SO_2_, NO_2_, or Ox) did not change the results substantially, we did not include these in the models.

Because the effects of AD can persist over several days, we examined the effects with several lag days (the case day [lag 0], lag 1, lag 2, lag 3, lag 4, and lag 5) in a single lag model. We also examined the cumulative lagged effects from day lag 0 to 1 (designated lag 01), lag 02, lag 03, lag 04, and lag 05.

All analyses were performed using STATA ver. 12.1 (StataCorp, College Station, TX, USA).

## Results

### Characteristics of the study participants from the two study areas

Table 1 shows the baseline characteristics of the study participants from the two study areas (Goto Island [*n* = 37] and the south Nagasaki area [*n* = 73]). A total of 56.8% in Goto Island and 61.6% in the south Nagasaki area were boys, respectively. The mean age, height, and weight of the participants in the Goto Islands and south Nagasaki area were 9.2 and 8.3 years, 133.1 and 127.6 cm, and 33.6 and 28.1 kg, respectively. Mild asthma cases predominated in the Goto Islands (47.2%) and moderate cases predominated in the south Nagasaki area (60.6%). The most commonly used medications in both areas were leukotriene receptor antagonists. The children in the Goto Islands spent an average of 2.4 h outdoors on weekdays and 3.1 h on weekends; the corresponding figures for the south Nagasaki area were 2.2 h and 2.6 h. Twenty-two (62.9%) children in the Goto Islands and thirty (41.1%) children in the south Nagasaki area lived with smokers. The observation period did not vary among areas (*p* value for χ^2^ test = 0.295).

**Table 1:** Baseline characteristics of participants between 2014 and 2016

	Goto Islands (*n* = 37)	South Nagasaki (*n* = 73)
Sex		
Male, *n* (%)	21 (56.8)	45 (61.6)
Female, *n* (%)	16 (43.2)	28 (38.4)
Age		
Mean age (years) (SD)	9.2 (3.3)	8.3 (2.5)
Body height		
Mean height (cm) (SD)	133.1 (18.3)	127.6 (15.3)
Body weight		
Mean weight (kg) (SD)	33.6 (14.2)	28.1 (10.5)
Severity of asthma		
Intermittent, *n* (%)	4 (11.1)	0 (0)
Mild, *n* (%)	17 (47.2)	19 (28.8)
Moderate, *n* (%)	9 (25.0)	40 (60.6)
Severe, *n* (%)	6 (16.7)	7 (10.6)
Long term medications*		
LTRA, *n* (%)	34 (91.2)	52 (71.2)
ICS (low), *n* (%)	11 (29.7)	20 (27.4)
ICS (medium), *n* (%)	13 (35.1)	35 (47.9)
ICS (high), *n* (%)	2 (5.4)	7 (9.6)
LABA (medium), *n* (%)	1 (2.7)	8 (10.9)
LABA (high), *n* (%)	1 (2.7)	0 (0)
Anti-histamine, *n* (%)	4 (10.8)	5 (6.8)
IgE		
Total serum IgE, IU/mL (SD)	643.9 (1081.1)	1277.2 (1975.6)
Daily outside activities		
Weekdays (hour) (SD)	2.4 (1.3)	2.2 (0.9)
Weekends (hour) (SD)	3.1 (1.5)	2.6 (1.6)
Using an air cleaner		
Every day, *n* (%)	12 (35.3)	26 (35.6)
Sometimes, *n* (%)	9 (26.5)	11 (15.1)
Never, *n* (%)	13 (38.2)	36 (49.3)
Secondhand smoking		
Smokers in home, *n* (%)	22 (62.9)	30 (41.1)
Observation period (years)		
Three, *n* (%)	13 (32.4)	37 (50.7)
Two, *n* (%)	12 (32.4)	19 (26.0)
One, *n* (%)	12 (35.1)	17 (23.3)

*LTRA* Leukotriene receptor antagonist, *ICS* Inhaled corticosteroids, *LABA* Long-acting β2 agonists

*Some of these medications are overlapped

### Number of AD days

**Fig. 2:**
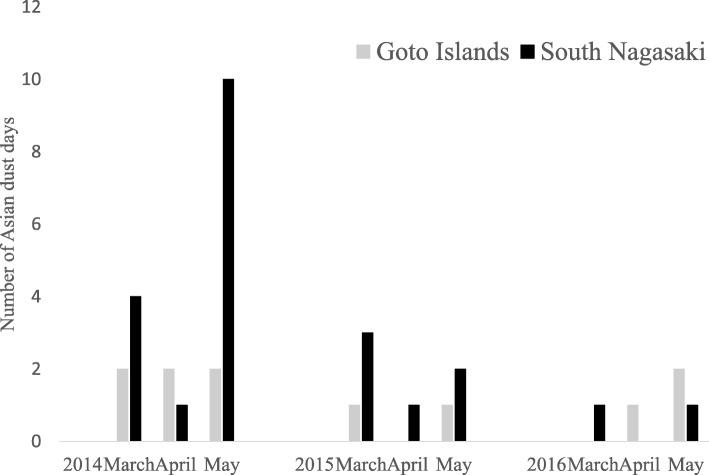
Number of Asian dust days in the study period

On the basis of our definition, the total number of AD days during the study period was 11 in the Goto Islands and 23 in the south Nagasaki area (Fig. 2). The largest number of AD days during the study period was observed in 2014. There was little difference in the number of AD days between Fukue Island and Nagasaki, except in May 2014 when LIDAR data were partly missing.

### AD, other air pollutants, and meteorological data

We compared the levels of AD, other air pollutants, and meteorological data on AD days and non-AD days during the months of March through May in 2014–2016 (Table 2). The mean AD coefficients on AD days (0.11/km in the Goto Islands, 0.07/km in the south Nagasaki area) were significantly higher than those on non-AD days (0.03/km in the Goto Islands, 0.04/km in the south Nagasaki area). Differences were also observed in the concentrations of other air pollutants and meteorological variables, except for NO2, temperature, and relative humidity.

**Table 2:** Summary of data on Asian dust, other air pollutants and meteorological factors (March 2014–May 2016)

	Goto Islands							South Nagasaki						
	Asian dust days (*n* = 11)			Non-Asian dust days (*n* = 265)				Asian dust days (*n* = 23)			Non-Asian dust days (*n* = 253)			
	Mean	SD	Median	Mean	SD	Median	*p* value^a,b^	Mean	SD	Median	Mean	SD	Median	*p* value^a,b^
Asian dust extinction coefficients (/km)	0.11	0.09	0.05	0.03	0.03	0.02	< 0.01^a^	0.07	0.04	0.05	0.04	0.03	0.03	< 0.01^a^
Spherical extinction coefficients (/km)	0.21	0.16	0.19	0.13	0.11	0.09	0.02^a^	0.17	0.14	0.13	0.14	0.07	0.12	0.35^a^
SO_2_ (ppb)	1.45	0.67	1.40	1.19	0.94	1.00	0.11^a^	1.90	0.83	1.70	1.34	0.88	1.15	< 0.01^a^
NO_2_ (ppb)	4.32	1.79	3.50	4.19	1.82	3.90	0.84^a^	7.02	2.00	7.50	6.68	2.34	6.20	0.50^a^
Ox (ppb)	58.5	9.24	58.2	51.6	9.69	51.2	0.02^a^	72.8	14.9	75.0	62.1	12.0	60.5	0.03^b^
SPM (μg/m^3^)	37.1	12.6	36.3	22.5	11.9	20.1	< 0.01^a^	42.9	18.4	36.9	23.4	9.4	22.1	< 0.01^a^
Average temperature (°C)	15.3	3.59	16.5	15.2	4.07	15.9	0.94^a^	17.3	3.9	18.8	15.8	4.4	16.6	0.11^a^
Maximum temperature (°C)	19.7	3.45	20.6	19.4	4.36	20.0	0.89^a^	21.7	4.1	22.3	20.2	4.7	20.8	0.17^a^
Minimum temperature (°C)	10.7	4.70	11.7	10.8	4.47	11.4	0.95^a^	13.2	4.3	14.6	12.0	4.6	12.8	0.18^a^
Humidity (%)	73.8	8.12	73.0	75.1	12.7	75.0	0.59^a^	67.0	10.9	67.0	69.9	12.4	69.0	0.27^b^

*p* value for ^a^Mann-Whitney *U* test and ^b^*t* test

Data on other air pollutants (SO_2_, NO_2_, Ox, SPM) in Nagasaki were calculated daily average concentrations from Isahaya city

### Study outcomes

Table 3 shows the incidence of asthma attacks and clinical symptoms. PEFRs were measured in 5996 days in the Goto Islands and 13,613 days in south Nagasaki. The largest number of PEFR reduction in the Goto Islands occurred in 2016, although the number of respiratory symptoms was highest in 2015. By contrast, the largest number of asthma attacks in Nagasaki occurred in 2014, and this year also saw the largest number of respiratory symptoms. According to asthma diary records, nose-related symptoms were more prevalent than those skin or eye symptoms. In both study areas, the largest number of days on which nose-related symptoms were reported was in 2015.

**Table 3:** Incidence of asthma attacks and clinical symptoms

	Goto Islands			South Nagasaki		
	2014	2015	2016	2014	2015	2016
Number of PEFR measurements						
Total peak flow days	2242 (100)	2023 (100)	1731 (100)	4286 (100)	4649 (100)	4678 (100)
Asthma attack defined by PEFR reduction*						
Mild (person day) (%)	428 (19.1)	358 (17.7)	554 (32.0)	1080 (25.2)	1011 (21.8)	729 (15.6)
Moderate (person day) (%)	115 (5.1)	158 (7.8)	232 (13.4)	289 (6.7)	309 (6.7)	216 (4.6)
Severe (person day) (%)	44 (1.9)	73 (3.6)	58 (3.4)	59 (1.4)	68 (1.5)	62 (1.3)
Self-reported respiratory symptoms						
Self-reported attacks						
0: None	2183 (87.9)	1802 (81.6)	2015 (91.3)	4156 (88.6)	4800 (91.5)	4910 (92.0)
1: Attack without dyspnea	221 (8.9)	339 (15.4)	92 (4.2)	457 (9.7)	379 (7.2)	329 (6.2)
2: Attack with little dyspnea	68 (2.7)	60 (2.7)	99 (4.5)	75 (1.6)	59 (1.1)	82 (1.5)
3: Attack with much dyspnea	12 (0.5)	7 (0.3)	2 (0.1)	3 (0.1)	6 (0.1)	15 (0.3)
Attack in exercise (person day) (%)	33 (1.3)	69 (3.1)	9 (0.4)	47 (1.0)	165 (3.2)	82 (1.5)
Sleep disturbance (person day) (%)	22 (0.9)	19 (0.9)	21 (1.0)	24 (0.5)	24 (0.5)	37 (0.7)
The usage of the inhalants (person day) (%)	110 (4.4)	159 (7.2)	79 (3.6)	276 (5.9)	152 (2.9)	231 (4.3)
The usage of the oral medicine (person day) (%)	69 (2.8)	200 (9.1)	29 (1.3)	178 (3.8)	141 (2.7)	155 (2.9)
Emergency visits (person day) (%)	14 (0.6)	23 (1.0)	11 (0.5)	11 (0.2)	17 (0.3)	20 (0.4)
Either symptoms (person day) (%)**	401 (16.1)	604 (27.4)	263 (11.9)	730 (15.6)	571 (10.9)	637 (11.9)
Self-reported other symptoms						
Skin (person day) (%)	93 (3.7)	375 (17.0)	146 (6.6)	573 (12.2)	742 (14.2)	442 (8.3)
Eye (person day) (%)	371 (14.9)	283 (12.8)	188 (8.5)	493 (10.5)	463 (8.8)	444 (8.3)
Nose (person day) (%)	562 (22.6)	863 (39.1)	699 (31.7)	1020 (21.7)	1310 (24.9)	1002 (18.8)
Either symptoms (person day) (%)***	797 (32.1)	1044 (47.3)	793 (35.9)	1390 (29.6)	1875 (35.8)	1460 (27.3)

*Base on the personal best peak expiratory flow rate of every month

**Secondary outcome related to respiratory symptoms

***Secondary outcome related to other than respiratory symptoms

**Fig. 3:**
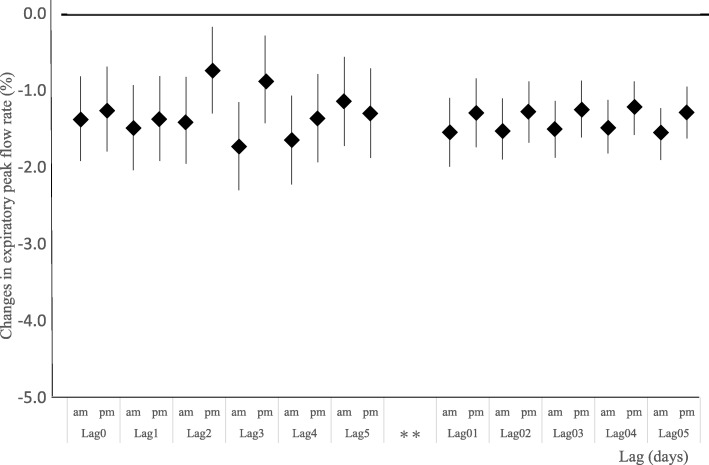
Association between Asian dust exposure and changes in peak expiratory flow rate (continuous variable) using a linear mixed-effects model adjusting for age, sex, daily mean temperature, daily relative humidity, and daily concentration of Ox. Single lag effects (left) and cumulative lag effects (right). The vertical bars represent 95% confidence intervals

The association between PEFR changes and AD days was consistent from lag 0 through lag 5 after adjusting for age, sex, temperature, and daily Ox (Fig. 3), with a 1.1–1.7% decrease in PEFR observed in the mornings and a 0.7–1.3% decreased observed in the evenings.

**Fig. 4:**
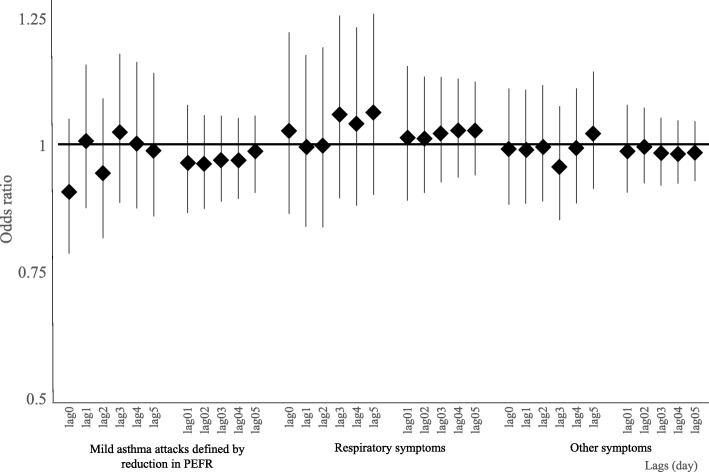
Association between Asian dust exposure and asthma attack and clinical symptoms (categorical outcomes) using conditional logistic regression models adjusting for temperature and relative humidity. The vertical bars represent 95% confidence intervals. Mild asthma attack was defined when a reduction of PEFR was below 80%. Respiratory symptoms included cough, wheezing, shortness of breath, asthma attacks in exercise, and sleep disturbance due to asthma attacks. Other symptoms included symptoms affecting the eyes, skin, and nose

The ORs for mild asthma attacks (less than 80% of the monthly maximum value) and respiratory and other symptoms are shown in Fig. 4. AD days were not associated with mild asthma attacks There was no evidence for the associations with moderate or severe asthma attacks (data not shown). Moreover, AD days were not associated with respiratory or other symptoms regardless of lag time.

## Discussion

We found that exposure to AD was consistently associated with the decrease in PEFR, although these changes were relatively small and clinically negligible. We found no clear association between AD and clinical symptoms, and asthma attacks defined by PEFR reductions.

Although adverse health effects related to AD exposure have been reported in children, the extent of these effects remains unclear. We reported in 2016 that exposure to AD was associated with increased numbers of emergency room visits by schoolchildren due to bronchial asthma attacks occurring 3 to 4 days after exposure [15]. Reduced PEFRs would be expected prior to emergency room visits due to bronchial asthma attacks. In a study involving daily PEFR measurements in schoolchildren, Hong et al. reported that outdoor particulate concentrations during periods when AD was present were significantly associated with reduced PEFR in asthmatic children but not in non-asthmatic children [16]. Watanabe et al. also reported significantly decreased PEFR in Japanese schoolchildren from day 0 to day 3 following AD exposure, although the effect size differed from year to year [21]. The mechanism through which AD affects pulmonary function is not yet fully understood. AD particles contain rock-forming minerals, such as quartz and feldspar, along with argillites, such as mica, kaolinite, and green mudstones. However, they also contain sulfur oxidants, nitrogen oxidants, ammonium, and microorganisms, all of which may contribute to such adverse health effects as allergic reactions and inflammation of the bronchi.

We hypothesized that children with bronchial asthma would show reduced PEFR following exposure to AD. A previous study in the Netherlands failed to find consistent positive or negative associations between respiratory symptoms and decreased peak flow [22]. The association we detected was so small that it was clinically negligible and no AD-related respiratory symptoms were recorded among our participants. A potential explanation for our negative findings is that the participants’ asthma was well controlled. We assumed that asthmatic children would be more vulnerable to AD exposure than non-asthmatic children because of their susceptibility and low threshold response to environmental changes. However, the participants in our study were taking medications for asthma, which prevented or controlled their clinical symptoms and asthma attacks even when exposed to AD. Furthermore, they may have been instructed by their parents to remain indoors on AD days as much as possible, which would have minimized their exposure.

One of the strengths of the present study was the continuous measurement of PEFR in the morning and evening during a 3-month period (March through May) over the three study years. In addition, the participating children measured their PEFR for 1 month before the study started. The PEFR measurements taken during the practice period tended to be lower than those taken during the study period and they tended to increase as the study progressed. This suggested that the children became increasingly skilled in performing PEFR measurements over the study period. We initially defined each participant’s highest PEFR as the highest value recorded during the study period. However, since the highest values tended to be recorded in the latter part of the study period, we switched to using the highest individual measurements for each month.

Another strength of the study was our use of LIDAR to assess AD exposure. In Japan, AD days are generally identified on the basis of visual observations by the Japan Meteorological Agency. These observations are made every hour, and an AD day is recorded when AD is observed for at least 1 hour on that day [23]. In contrast, LIDAR measurements allow quantitative evaluation of AD particle levels to distinguish between AD days and non-AD days. We must acknowledge, however, that in May 2014, there was a difference in the number of AD days between Goto Islands and Nagasaki because some data were missing.

A potential limitation of this study was the possibility of PEFR measurement errors. PEFR measurement is highly effort-dependent and variability is significant even among those with experience in taking PEFR measurements. Therefore, there is a possibility that the association between AD exposure and PEFR changes in this study was underestimated.

Another limitation might be an exposure misclassification. For instance, the LIDAR in Omura City was located approximately 30 km from the center of Nagasaki City. This misclassification is non-differential, which leads to a bias towards the null.

## Conclusions

Our study showed that exposure to AD was associated with reduced PEFRs in asthmatic children, although the changes were not large enough to induce clinically apparent symptoms. Appropriate medical management of asthmatic children may be necessary to avoid adverse health effects caused by AD.

## Data Availability

The datasets generated and analyzed during the current study are not publicly available because we did not receive consents for data provision to the third party.

## Abbreviations

AD: Asian dust
CIs: Confidence intervals
LIDAR: Light detection and ranging
NO_2_: Nitrogen dioxide
ORs: Odds ratios
Ox: Photochemical oxidants
PEFRs: Peak expiratory flow rates
SO_2_: Sulfur dioxide
